# Dental Composite Performance Prediction Using Artificial Intelligence

**DOI:** 10.1177/00220345241311888

**Published:** 2025-02-14

**Authors:** K. Paniagua, K. Whang, K. Joshi, H. Son, Y.S. Kim, M. Flores

**Affiliations:** 1 Department of Electrical and Computer Engineering, the University of Texas at San Antonio, San Antonio, TX, USA; 2 Department of Comprehensive Dentistry, the University of Texas Health Science Center at San Antonio, San Antonio, TX, USA; 3 Department of Oral & Maxillofacial Surgery, School of Dentistry, University of Texas Health Science Center at San Antonio, San Antonio, TX, USA; 4 Programs in Integrated Biomedical Sciences, Translational Sciences, Biomedical Engineering, Radiological Sciences, University of Texas Health Science Center at San Antonio, San Antonio, TX, USA

**Keywords:** AI, machine learning, dental composites, forecasting, composite attributes, polymerization shrinkage

## Abstract

There is a need to increase the performance and longevity of dental composites and accelerate the translation of novel composites to the market. This study explores the use of artificial intelligence (AI), specifically machine learning (ML) models, to predict the performance outcomes (POs) of dental composites from their composite attributes. A comprehensive dataset was carefully curated and refined from 200+ publications. Nine ML models were trained to predict discrete POs, and their performance was evaluated. Five models were used for regression analysis of continuous POs. Different ML models performed better on different POs. The k-nearest neighbors (KNN) model excelled in predicting flexural modulus (FlexMod), Decision Tree model in flexural strength (FlexStr) and volumetric shrinkage (ShrinkV), and Logistic Regression and Support Vector Machine models in shrinkage stress (ShrinkStr). Receiver-operating characteristic area under the curve analysis confirmed these results but found that Random Forest was more effective for FlexStr and ShrinkV, suggesting the possibility of Decision Tree overfitting the data. Regression analysis revealed that the voting regressor was superior for FlexMod and ShrinkV predictions, while Decision Tree Regression was optimal for FlexStr and ShrinkStr. Feature importance analysis indicated triethylene glycol dimethacrylate is a key contributor to FlexMod and ShrinkV, bisphenol A glycidyl dimethacrylate and urethane dimethacrylate to FlexStr, and depth of cure, degree of monomer-to-polymer conversion, and filler loading to ShrinkStr. There is a need to conduct a full analysis using multiple ML models because different models predict different POs better and for a large, comprehensive dataset to train robust AI models to facilitate the prediction and optimization of composite properties and support the development of new dental materials.

## Introduction

Direct dental composites are widely used in restorative dentistry for the treatment of cavities and defects in teeth. These materials are composed of a resin matrix and fillers, and their properties, such as strength and wear resistance directly affect their performance and longevity in the oral cavity. Given the variety of filler types, resin compositions, and other material characteristics, it can be challenging to predict how a specific composite will perform in clinical conditions. Recent advancements in machine learning (ML) have opened new possibilities for predicting the properties of dental composites based on their composition and processing conditions. ML models can learn from datasets of composite formulations and their corresponding performance outcomes to identify patterns and make predictions about future material behavior. This can significantly accelerate the development of improved composite materials, provide insights for clinical decision-making, and enhance patient outcomes. To date, it is known that amalgam still outperforms composites in longevity (Araujo et al. 2019) with a success rate of 92% over 55 mo compared with 85% for composites and composite restorations lasting <8 y (Van Nieuwenhuysen et al. 2003; Beck et al. 2015; Moraschini et al. 2015). The main causes of composite failure include composite fracture and recurrent caries. This can be related to composite polymerization shrinkage stress, resin degradation, and mechanical properties (Mjör et al. 2000; Van Nieuwenhuysen et al. 2003). Therefore, developing advanced composites is a critical need.

Although research into novel composites is ongoing (Satsangi et al. 2005; Danso et al. 2018; Whang et al. 2019; Sathissarat et al. 2021; Bergeron et al. 2022; Liao et al. 2022; Sun et al. 2022; Usul et al. 2022), current commercial composites use the classic combination of bisphenol A glycidyl dimethacrylate (BisGMA), ethoxylated bisphenol A dimethacrylate (BisEMA), urethane dimethacrylate (UDMA), triethylene glycol dimethacrylate (TEGDMA), and glass filler due to the time, effort, and expense of developing new systems and gaining Food and Drug Administration approval. Designing an optimal composite is challenging because of the nonlinear relationship between composition and properties.

Machine and deep learning models from artificial intelligence (AI) offer powerful tools for nonlinear classification in computational material science (Flores et al. 2022). So far, only one AI study has explored composite materials, focusing on the effect of composite attributes (CAs) on the flexural strength of computed-aided design/computed-aided manufacturing resin composite blocks. UDMA, TEGDMA, and filler content were most important in predicting strength with high prediction performance and low error (Li et al. 2022). However, limitations included a small sample size that may have led to overfitting of the data and artificially high predictive performance and a narrow focus on monomer type, filler type, and filler loading. Furthermore, this model may not be as useful for direct composites, which have many more variables. There is a wide range of performance outcome (Usul et al. 2022) values for the same direct dental composite, and other POs need to be assessed, as strength is not the only important PO, to provide a clearer understanding of how effective AI is in identifying the most important CAs and optimizing composite design.

Thus, the overall objective of this study is to investigate the efficacy of AI models in predicting POs to develop more durable composites while reducing the time, work, and expense needed to translate experimental composites to the market. Specifically, we aim to build a comprehensive dental composite dataset and determine which AI models best predict which CAs are related to specific composite POs.

## Materials and Methods

### Dataset Construction

Google Scholar and company brochures were used to create a database of CAs and POs. CAs included monomer type, concentration and molecular weight, initiator type and concentration, degree of monomer-to-polymer conversion, filler type, loading, shape and size, viscosity, density, and index of refraction. Composite POs included depth of cure, radiopacity, flexural modulus (FlexMod), flexural strength (FlexStr), compressive strength (CompStr), fracture toughness (FracTough), fracture work (FrackWk), polymerization volumetric shrinkage (ShrinkV), shrinkage stress (ShrinkStr), wear depth and number of cycles, water sorption and solubility, survival rate and length, in vitro cytotoxicity (ED_50_), total fluoride release and duration, and remineralization potential.

### Data Preprocessing

All string values were changed to integer or floats. Samples lacking the target POs and CAs with only zeros were excluded. Missing CA values were imputed using the k-nearest neighbors (KNN) algorithm imputation for regression analysis and multivariate imputation for classification (Troyanskaya et al. 2001) to maximize usable data and ensure robust results. Because the data are continuous, each PO datum was divided into 2 classes (high/low) with respect to its mean value. Low means the PO value is smaller or equal to the mean value, and high means it is larger.

### AI Analysis

Our dataset contains 233 samples, with considerable variation in the number of available entries for each target column. For example, flexural modulus (FlexMod) has 103 entries, while fracture work (FracWk) has only 9 entries. In ML, datasets with fewer than a few hundred samples, particularly when target columns have significant gaps in coverage, are generally considered small. This limited sample size can lead to high variance and overfitting issues, especially with complex models. So feature engineering was used to extract meaningful features (Zien et al 2009), simple ML models that work with little data were used to prevent overfitting, and evaluation tools were implemented to assess the model’s performance.

#### Classification analysis

Nine ML classification algorithms (support vector machine [SVM], Decision Tree, KNN classifier, Light Gradient Boosting Machine [LGBM], Random Forest, Logistic Regression, Gaussian Naïve Bayes, Extreme Learning Machine [ELM], and Extreme Grading Boosting [XGBoost]) were trained using 80% of the data and tested on the remaining 20% of the data to predict each composite PO.

## Results

### Dataset Construction

From 200+ publications, 321 composite formulations with 28 CAs and 17 POs were included. Because experimental composites tended to use unique CAs that were not widely used, limiting the utility of that data, only commercial composites were included to evaluate the ML models. After that reduction, POs with no values were removed, and CompStr, FracTough, and FracWrk, which had perfect performance scores due to small samples sizes and overfitting, were also removed, reducing the size of the final curated dataset to 233 composites with 17 CAs (Appendix Table 1) and 7 POs (Appendix Table 2). Missing CA values were imputed to ensure the model accurately captured patterns and relationships without bias from missing data. Composites with no values for specific POs were also removed, resulting in different samples sizes (Appendix Table 2). FlexStr had the largest sample size (54.9%) and FrackWk the smallest (3.9%). Small sample sizes led to overfitting for POs, so CompStr, FracTough, and FracWk were excluded from further analysis. This highlights the need for a larger dataset of composites to train more robust models.

### Classification Analysis

#### Flexural modulus

**Figure 1.**
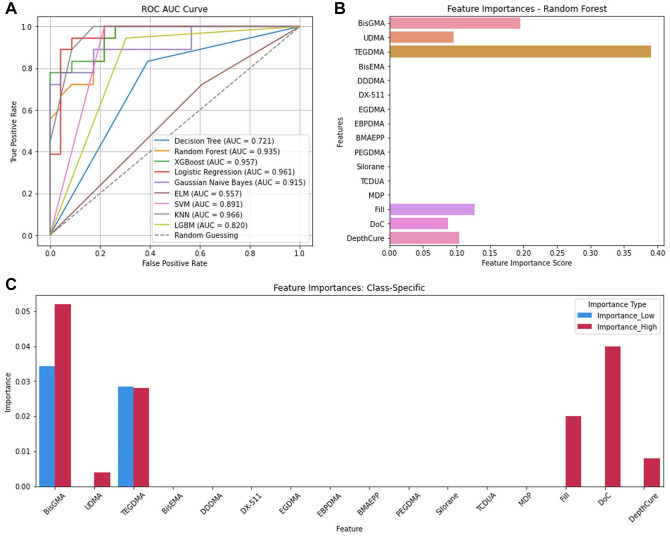
Flexural modulus. (**A**) ROC AUC curve of the 9 algorithms. (**B**) Feature importances for flexural modulus. (**C**) Feature importances per class. AUC, area under the curve; ROC, receiver-operating characteristic.

The KNN model was best in predicting FlexMod classification (accuracy = 0.90, precision = 0.92, recall score = 0.90, F1 score = 0.90; Appendix Table 3). KNN was balanced and accurate in identifying low and high FlexMod instances while minimizing false positives. KNN also achieved the highest area under the curve (AUC) score (0.97; Fig. 1A), confirming the model’s strength to predict FlexMod across different thresholds.

Feature importance analysis using the Random Forest model showed that the top 6 CAs affecting FlexMod were TEGDMA (Feature Importance Score [FIS] = 0.39), BisGMA (0.19), Fill (0.13), DepthCure (0.10), UDMA (0.09), and DoC (0.08) (Fig. 1B, Appendix Table 3). Thus, TEDGMA had the greatest influence on FlexMod, followed by BisGMA. Other CAs had negligible importance, likely because fewer composites contained those CAs.

Permutation importance scores showed that for predicting low FlexMod, TEGDMA caused the largest accuracy drop (4.8%), followed by BisGMA (Permutation Score [PS] = 0.04) and UDMA (0.008). For high FlexMod, TEGDMA was most important (0.056), followed by BisGMA (0.048), DoC (0.048), and Fill (0.020). While some scores were low, they remained significant for overall predictions. For example, when both BisGMA and TEGDMA were permuted, the accuracy dropped by 20%. Thus, these CAs need to be controlled in composite design.

#### Flexural strength

**Figure 2.**
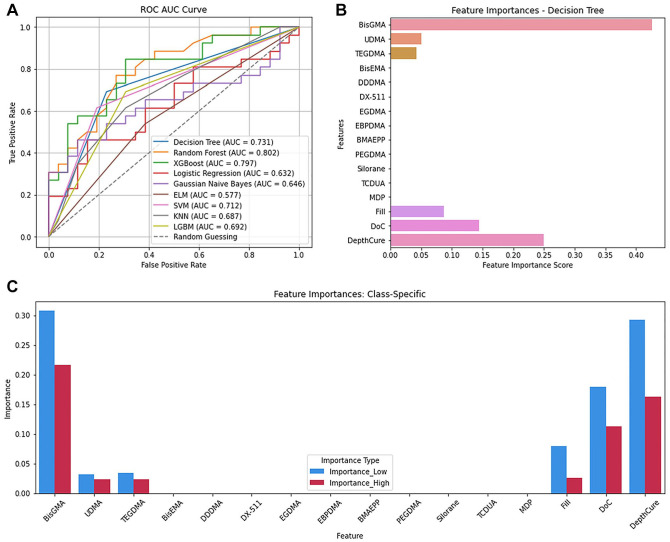
Flexural strength. (**A**) ROC AUC curve of the 9 algorithms. (**B**) Feature importances for flexural strength. (**C**) Feature importances per class. AUC, area under the curve; ROC, receiver-operating characteristic.

For classifying FlexStr, Decision Tree performed best (Appendix Table 4) with accuracy, precision, recall, and F1 scores of 0.73 each. However, it ranked third in AUC (0.73) (Fig. 2A). Random forest (0.802) and XGBoost (0.797) better distinguished low and high FlexStr across thresholds.

Feature importance analysis using the Decision Tree model (Fig. 2B) revealed that the important CAs for predicting FlexStr were BisGMA (0.42), DepthCure (0.25), DoC (0.14), Fill (0.08), UDMA (0.05), and TEGDMA (0.04). BisGMA was most important for low FlexStr (31% accuracy drop) when permuted, followed by DepthCure (PS = 0.31), DoC (0.19), Fill (0.08), TEGDMA (0.034), and UDMA (0.031) (Fig. 2C). For high FlexStr, BisGMA was the most important (0.22), followed by DepthCure (0.16), DoC (0.11), Fill (0.026), TEGDMA (0.024), and UDMA (0.023).

#### Compressive strength and fracture toughness

For CompStr and FracTough, the Decision Tree and Random Forest models overfitted the data (scores = 1.0) due to the limited sample sizes (Appendix Tables 5 and 6). For example, for CompStr, of the 17 CAs, only Fill (37 of 42 samples), DoC (11) and DepthCure (4) had values. Such limited datasets lead to poor generalization and overfitting, and imputing missing values could introduce bias, resulting in unreliable models. Thus, further analyses were abandoned.

#### Volumetric shrinkage

**Figure 3.**
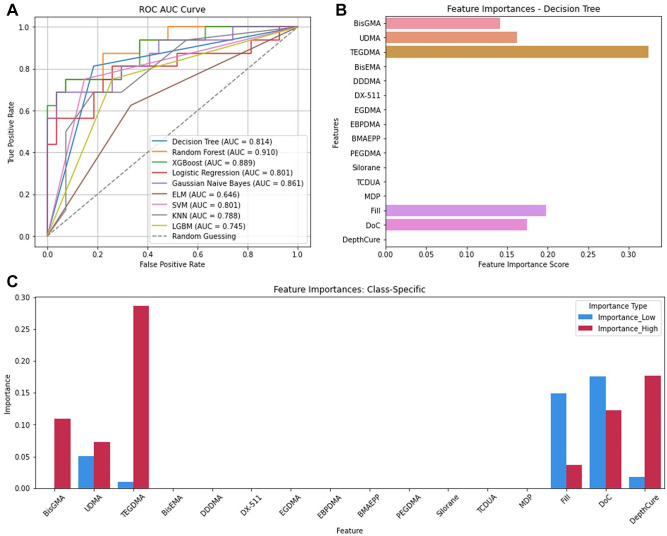
Volumetric shrinkage. (**A**) ROC AUC curve of the 9 algorithms. (**B**) Feature importances for volumetric shrinkage. (**C**) Feature importances per class. AUC, area under the curve; ROC, receiver-operating characteristic.

The Decision Tree model predicted ShrinkV best (accuracy = 0.81, precision = 0.82, recall = 0.81, F1 = 0.82; Appendix Table 7) but it ranked fourth in AUC (0.81) (Fig. 3A). Random forest achieved the highest AUC (0.910), followed by XGBoost (0.889) and Gaussian Naïve Bayes (0.861), indicating that while Decision Tree performed well overall, other models were superior in distinguishing between low and high ShrinkV across various thresholds. Feature importance analysis with Decision Tree showed that the most important CAs for predicting ShrinkV (Fig. 3B) were TEGDMA (FIS = 0.32), Fill (0.19), DoC (0.17), UDMA (0.16), and BisGMA (0.14). For low ShrinkV, DoC (PS = 0.17), Fill (0.14), UDMA (0.05), DepthCure (0.17), and TEGDMA (0.01) were most important. For high ShrinkV, TEGDMA (0.28), DepthCure (0.18), DoC (0.12), BisGMA (0.11), UDMA (0.07), and Fill (0.04) were most important (Fig. 3C).

#### Shrinkage stress

**Figure 4.**
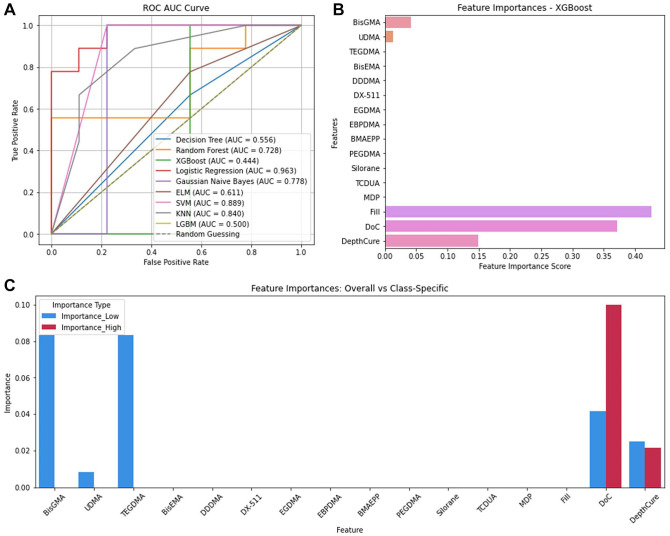
Shrinkage stress. (**A**) ROC AUC curve of the 9 algorithms. (**B**) Feature importances for shrinkage stress. (**C**) Feature importances per class. AUC, area under the curve; ROC, receiver-operating characteristic.

Logistic regression, SVM, and XGBoost predicted ShrinkStr best (accuracy = 0.89, precision = 0.91, recall = 0.89, F1 = 0.89; Appendix Table 8). Logistic regression also had the highest AUC (0.963), followed by SVM (0.899). However, XGBoost had an AUC of only 0.444 (Fig. 4A). Feature importance analysis (Fig. 4B), Fill (FIS = 0.43), DoC (0.37), DepthCure (0.15), BisGMA (0.04), and UDMA (0.01) best predicted ShrinkStr. BisGMA and TEGDMA were most important for low ShrinkStr (PS = 0.08), followed by DoC (0.04), DepthCure (0.03), and UDMA (0.008) (Fig. 4C). For high ShrinkStr, DoC was most important (0.10), followed by DepthCure (0.02).

#### Regression

**Figure 5.**
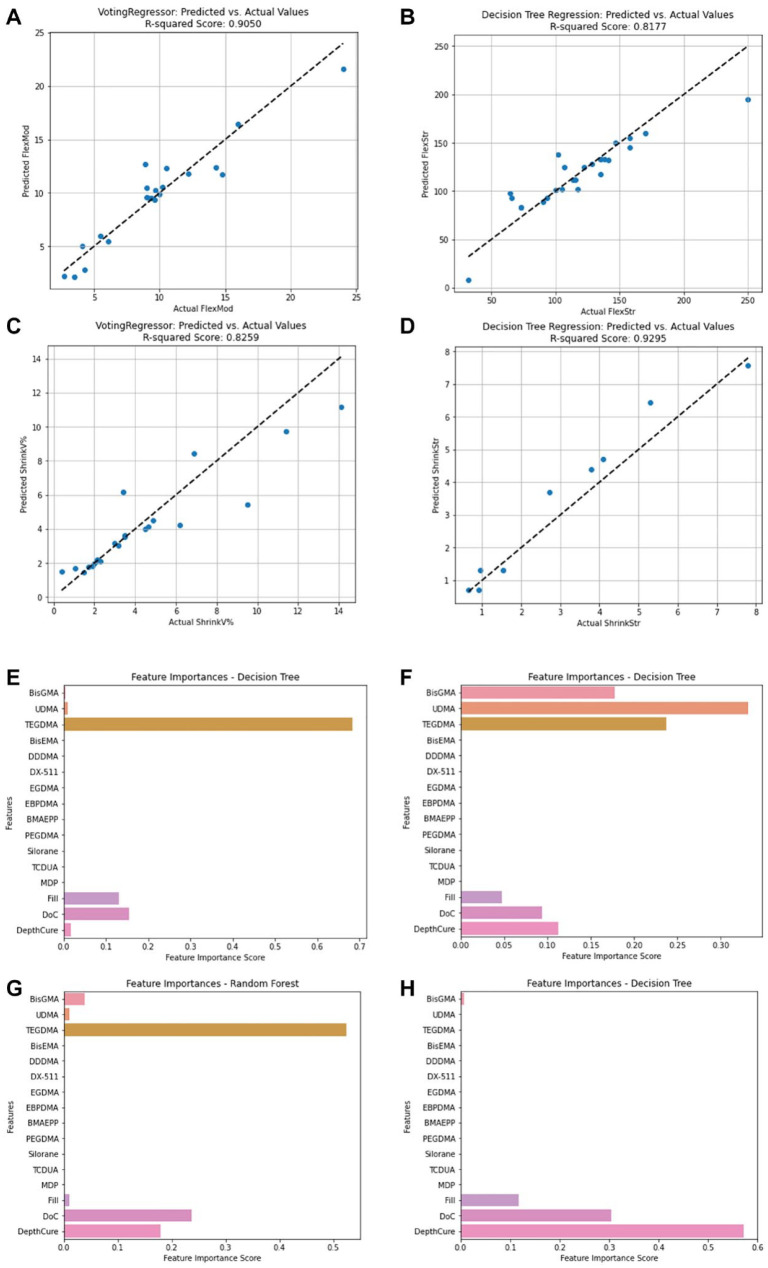
Model fit and feature importances. Predicted versus actual values from (**A**) Voting Regressor for FlexMod, (**B**) Decision Tree for FlexStr, (**C**) Voting Regressor for ShrinkV, and (**D**) Decision Tree for ShrinkStr. Feature importances for (**E**) FlexMod, (**F**) FlexStr, (**G**) ShrinkV, and (**H**) ShrinkStr. FlexMod, flexural modulus; FlexStr: flexural strength; ShrinkStr, shrinkage stress; ShrinkV, volumetric shrinkage.

The voting regressor model excelled in predicting ShrinkV and FlexMod (*R*^2^ = 0.83 and 0.91, respectively; Appendix Table 9). The Decision Tree regression model performed best for FlexStr and ShrinkStr (*R*^2^ = 0.82 and 0.93) with most data points clustering around the ideal (dotted) line, indicating close alignment between predicted and actual values for the 4 POs analyzed (Fig. 5C).

Overall, ShrinkStr had the lowest error metrics (mean absolute error [MAE] = 0.49 MPa (Willmott and Matsuura, 2005), mean square error [MSE] = 0.36 MPa, root mean square error [RMSE] = 0.6 MPa (Willmott and Matsuura, 2005), median absolute error = 0.34 MPa (Willmott and Matsuura, 2005), and max error [ME] = 1.15 MPa) (Dau et al., 2014), indicating that the model predicts ShrinkStr values close to the actual measurements (Appendix Table 10). However, for FlexStr, despite achieving a relatively high *R*^2^ score of 0.82, the error metrics had the highest discrepancies between predicted and actual values, underscoring the challenges in accurately predicting FlexStr using the current models. Model refinement or alternative approaches may be necessary to improve prediction accuracy. For algorithms with lower performance, detailed error scores are provided (Appendix Tables 11–14).

Feature importance analysis using the regression models for each of the 4 predicted POs showed that for FlexMod, TEGDMA (FIS = 0.68), DoC (0.15), and Fill (0.13) were most important (Fig. 5E). For FlexStr, UDMA (0.33), TEGDMA (0.23), and BisGMA (0.17) were key (Fig. 5F). For ShrinkV, TEGDMA (0.52), DoC (0.24), and DepthCure (0.18) were dominant (Fig. 5G). For ShrinkStr, DepthCure (0.57), DoC (0.30), and Fill (0.12) were most influential (Fig. 5H).

## Discussion

The objectives of this work were to build a comprehensive dataset of CAs and POs from the literature and use that to determine the efficacy of different ML models in predicting POs so we can optimize composite design and develop durable composites. Despite reviewing 200+ publications, after excluding unique composite systems that are not widely used, a final dataset of 233 commercial composite samples consisting of 17 CAs and 7 POs were analyzed with different ML models. This is substantially larger than the only published article on using AI to predict POs, which contained only 12 samples and potentially had overfitting of the data (Li et al. 2022). This was also evident in the present study for CompStr, FracTough, and FracWrk, which had perfect performance scores due to small samples sizes and overfitting. Thus, these POs were excluded from further analyses. While other AI applications have tens of thousands of samples, that is not true here. More dental composite data and the eventual inclusion of unique experimental composites is needed. A future goal is to create an open-source dataset, where researchers can contribute their research data in exchange for the use of the dataset for their research. Another way to mitigate the small sample size for unique monomers and fillers, and a future goal, is to use chemical formulas as CAs instead of simply monomer name and/or concentration.

For classified data, KNN predicted FlexMod best (Appendix Table 3, Fig. 1A), whereas Decision Tree excelled for FlexStr and ShrinkV (Appendix Tables 4, 7) and Logistic Regression and SVM for ShrinkStr (Appendix Table 8). KNN can detect local patterns by comparing the proximity of CA values within the same PO class. It is also nonparametric and handles complex, nonlinear relationships better without needing prior knowledge of the data distribution (Fix 1985).

Decision Tree splits data into subsets based on input features, leading to different class labels (Thomas et al. 2020). Despite high accuracy, precision, recall, and F1 scores, its AUC scores were lower (Figs. 2A, 3A), probably due to overfitting. FlexStr had 31 nodes and a depth of 6, while ShrinkV had 21 nodes and a depth of 9 (Appendix Figs. 2 and 3) (Bramer 2007), making the model too complex for the data size, and the model seems to have memorized the training data instead of identifying patterns, reducing its ability to distinguish between low and high FlexStr and ShrinkV. This lowers the true-positive rate across AUC thresholds, reducing the AUC score (Hoo et al., 2017).

Ensemble methods (Random Forest and XGBoost) improve predictions by aggregating results from multiple decision trees (Khan et al. 2023), identifying more robust patterns across AUC thresholds. Random Forest reduces variance and improves generalization by building trees from different data subsets (Breiman 2001), while XGBoost refines predictions through models that correct errors, using gradient descent for optimization and incorporating regularization to prevent overfitting (Noviandy et al. 2024). Gaussian Naïve Bayes assumes feature independence and performs well with less complex data (Ontivero-Ortega et al. 2017). Despite this, Decision Tree remains a good choice due to its interpretability. Each decision node represents a feature split, making predictions easy to understand.

For ShrinkStr, Logistic Regression, SVM, and XGBoost models excelled (Appendix Table 8), but only Logistic Regression and SVM had high AUC scores (Fig. 5A). Logistic Regression stands out in its probabilistic interpretation and simplicity, useful for understanding the feature impact (Ranganathan et al. 2017). SVM’s strength lies in its ability to handle complex, nonlinear relationships through kernel functions (Babu et al. 2024). XGBoost’s lower AUC suggests challenges with generalization or overfitting despite solid performance. In the regression analysis, the Voting Regressor performed well for FlexMod and ShrinkV, leveraging combined model predictions (Appendix Table 9, Appendix Figure 5). Decision Tree Regression demonstrated its capacity to capture complex relationships when predicting FlexStr and ShrinkStr (Appendix Table 9, Appendix Figure 5), because Decision Tree is good at modeling nonlinear relationships and interactions between CAs.

The variability in model performance across POs is due to the differing relationships between POs and CAs. For instance, POs with more straightforward or linear relationships with CAs may benefit from the probabilistic and direct nature of models such as the Voting Regressor. However, POs that involve more complex interactions fare better with models capable of handling nonlinear data, such as Decision Tree Regression (Dumont et al., 2009). Ensemble methods, such as Voting Regressor, excel at linear predictions (Appendix Tables 9 and 10), and the predictive power of Decision Tree Regression captures complex interactions across CAs (Appendix Tables 9 and 10).

Results for FlexStr demonstrated greater robustness and reliability compared with those of Li et al. (2022), due to our significantly larger sample size (128 versus 12), which improved the detection of patterns and relationships. Despite this, for both studies, Decision Tree had the highest *R*^2^ scores. Regardless, our results show that one model does not fit all POs because the relationship between CAs and POs may not be linear.

Appendix Table 11 summarizes feature importance analyses from the classification and regression analyses. Both identified TEGDMA as most important for FlexMod (Appendix Table 11, Figs. 1B, 5E) and ShrinkV (Appendix Table 11, Figs. 3B, 5G). TEGDMA’s low molecular weight increases crosslink density, raises the modulus, and increases polymerization shrinkage. Interestingly, it is not a major contributor to ShrinkStr, since increased ShrinkV generally leads to ShrinkStr. Nonetheless, composites with a higher depth of cure and filler loading tend to be in low-shrinkage bulk cure composites, so that may have overshadowed the effect of TEGDMA. BisGMA concentration was also important for FlexMod, ShrinkV, and ShrinkStr. BisGMA is a strong material, and with 2 benzene rings in each monomer, it can impart rigidity (FlexMod) to the composite. It is also a large monomer, so high concentrations of BisGMA would reduce shrinkage and potentially shrinkage stress. Similarly, inorganic filler loading will impart rigidity and reduce ShrinkV and ShrinkStr because glass filler particles are stronger than organic monomers, and they do not participate in the polymerization reaction and impart polymerization shrinkage. DoC would increase crosslinking, increase FlexMod, increase the number of double bonds being converted to single bonds, and increase the reduction of free space, thus increasing Shrink V and ShrinkStr. Finally, DepthCure’s prominence in ShrinkStr may reflect its association with bulk-fill composites, which have reduced ShrinkV and ShrinkStr, as does high Fill. Interestingly, BisGMA was less important in the regression for ShrinkV and ShrinkStr despite its importance in classified data. For FlexStr, BisGMA, DepthCure, DoC, Fill, UDMA, and TEGDMA were recognized, with BisGMA being the most important in classification and UDMA in regression (Appendix Table 11, Figs. 2B, 5F). This aligns with their roles as the largest and strongest monomer components. DoC and Fill are important for FlexStr, as explained above for FlexMod.

## Conclusion

The objectives of this work were to build a comprehensive dataset of dental composites from an extensive literature curation and evaluate the effectiveness of ML models to optimize the design of composites. This work marks the first attempt at generating an extensive dataset of dental CAs and POs. Our results show the need to extend this database to improve AI prediction and that different models are more effective in predicting different composite POs. Once more data are generated, AI models would allow researchers and manufacturers to input the CAs of experimental composites and use the best models to predict POs before fabrication, significantly reducing development time by narrowing the starting groups for testing.

## Author Contributions

K.P. Rivera, contributed to acquisition, analysis, and interpretation, drafted and critically revised the manuscript; K. Whang, M. Flores, contributed to conception, design, data acquisition, analysis, and interpretation, drafted and critically revised the manuscript; K. Joshi, contributed to data analysis, critically revised the manuscript; H. Son, Y.S. Kim, contributed to data acquisition and interpretation, critically revised the manuscript. All authors gave their final approval and agreed to be accountable for all aspects of the work.

## Supplemental Material

sj-docx-1-jdr-10.1177_00220345241311888 – Supplemental material for Dental Composite Performance Prediction Using Artificial Intelligence Supplemental material, sj-docx-1-jdr-10.1177_00220345241311888 for Dental Composite Performance Prediction Using Artificial Intelligence by K. Paniagua, K. Whang, K. Joshi, H. Son, Y.S. Kim and M. Flores in Journal of Dental Research
