# Supplementary material for: Dental Composite Performance Prediction Using Artificial Intelligence
Source: J Dent Res. 2025 Feb 14;104(5):513–21. doi: 10.1177/00220345241311888 (PMC12000627; doi:10.1177/00220345241311888)
Supplement: sj-docx-1-jdr-10.1177_00220345241311888 – Supplemental material for Dental Composite Performance Prediction Using Artificial Intelligence [file sj-docx-1-jdr-10.1177_00220345241311888.docx]

**Dental Composite Performance Prediction Using Artificial Intelligence**

Karla Paniagua Rivera^1^, Kyumin Whang^2^, Krishna Joshi^2^, Hyeonwi Son^3^, Yu Shin Kim^3,4^, Mario Flores^1^

^1^Department of Electrical and Computer Engineering, the University of Texas at San Antonio, San Antonio, TX, 78249, USA

^2^Department of Comprehensive Dentistry, the University of Texas Health Science Center at San Antonio, San Antonio, TX 78229, USA

^3^Department of Oral & Maxillofacial Surgery, School of Dentistry; ^4^Programs in Integrated Biomedical Sciences, Translational Sciences, Biomedical Engineering, Radiological Sciences, University of Texas Health Science Center at San Antonio, San Antonio, TX 78229, USA

***correspondence:**

Mario Flores; Address: One UTSA Circle, San Antonio, TX 78249; Phone:210-8433834; email: [mario.flores@utsa.edu](mailto:mario.flores@utsa.edu)

# **Materials and Methods**

Classification Analysis

Nine ML classification algorithms (Support Vector Machine (SVM), Decision Tree, KNN Classifier, Light Gradient Boosting Machine (LGBM), Random Forest, Logistic Regression, Gaussian Naïve Bayes, Extreme Learning Machine (ELM), and Extreme Grading Boosting (XGBoost)) were trained using 80% of the data and tested on the remaining 20% of the data to predict each composite PO.

For the Support Vector Machine classifier, various gamma values (kernel coefficient) were tested, ranging from 1 to 10 in increments of 1, and then in increments of 10 up to 100. The gamma value that achieved the highest classification accuracy was selected. Additionally, the regularization parameter was set to C=10 to prioritize correctly classifying all training examples, balancing flexibility and model complexity. The radial basis function (rbf) kernel was used for SVM, given its effectiveness for non-linear relationships in the data.

For the Extreme Learning Machine (ELM) classifier, 100 neurons were utilized, considering the small dataset size, with a sigmoid activation function (sigm) for the neurons.

For the Decision Tree, KNeighbors, LGBM, Random Forest, Logistic Regression, Gaussian Naïve Bayes, and XGBoost classifiers, default parameters were used, as preliminary tests indicated that these defaults performed sufficiently well for the given dataset.

Model performance was evaluated using accuracy (proportion of correctly classified instances), precision (proportion of true positive instances out of all instances predicted as positive), recall (proportion of true positives out of all actual positives), F1score (the harmonic mean of precision and recall, i.e. how well those two correlate), Receiver Operating Characteristic Area Under the Curve (ROC AUC) and AUC scores. The ROC AUC curve plots the true positive rate (sensitivity) against the false positive rate (1-specificity), and the higher the AUC scores the better the model’s performance (Hoo et al. 2017).

The data was further analyzed using the best performing model with a feature importance tool to rank the importance of features (Ontivero-Ortega et al.) in affecting the PO (Zien et al. 2009). The feature importance tool measures the contribution of each CA to the model’s predictions by evaluating how much each CA reduces impurity in the decision-making process. Impurity represents how mixed the data is at a particular node in the tree in terms of the PO’s class (Appendix Figure 1). Low impurity occurs when a node contains mostly one class, indicating more homogeneous and better-classified data.

Permutation importance was also determined, which randomly shuffles the values of each CA, observes its impact on the model’s accuracy and provides an estimate of its importance in the model’s accuracy in predicting low or high PO. So, while the feature importance shows the contribution of each CA in changing the PO, permutation importance measures how important the CA is for the model to correctly predict the classification of the PO.

Regression Analysis

Since the original data is continuous, five regression models (Support Vector Regression (SVR) (Chang and Lin 2011; Platt 1999), Decision Tree Regression (Dumont et al. 2009), Histogram Gradient Boosting Regression (HistGradientBoost) (Mayer et al. 2022), Random Forest Regression (Breiman 2001), and a voting regressor (an ensemble method that combines the predictions from multiple regression models to produce a final prediction with improved accuracy and robustness)) were trained on 80% of the data and tested on the remaining 20% to predict those continuous values. The voting regressor is composed of Linear, Random Forest, KNN, SVR, HistGradientBoost, and Decision Tree regression models.

The Voting Regressor utilizes a simple averaging approach for its ensemble strategy. In this model, predictions from Linear Regression, Random Forest Regressor, KNeighbors Regressor, Decision Tree Regressor, Support Vector Regressor, and Histogram-based Gradient Boosting Regressor are combined by calculating the average of each model’s predictions. In this strategy, each regressor contributes equally, without weighting, to the final prediction. This approach effectively reduces variance and improves prediction robustness by leveraging the strengths of diverse model types, thereby minimizing model-specific biases in the ensemble’s output.

The explained variance score, R^2^, was calculated to evaluate Regression model performance. It measures the influence of the CAs on the variance in the PO and quantifies how well the model accounts for the variability. Additionally, five different errors were calculated to see how well a model predicts the POs: Mean Absolute Error (MAE) provides an average of absolute errors (Willmott and Matsuura 2005), Mean Squared Error (MSE) emphasizes larger errors (Willmott and Matsuura 2005), Root Mean Squared Error (RMSE) gives a measure in the original units of the target variable (Willmott and Matsuura 2005), Median Absolute Error (MedAE) is robust against outliers (Aristodemou et al. 2015), and Max Error (ME) highlights the worst-case prediction error (Dau et al. 2014).

# **Appendix Tables**

**Appendix Table 1.** Composite Attributes (Cas), Sample Sizes and Proportions to All Samples.

| **CA** | **Sample Size** | **Proportion (%)** |
| --- | --- | --- |
| 1. BisGMA Concentration (wt %) | 39 | 17 |
| 1. UDMA Concentration (wt %) | 11 | 5 |
| 1. TEGDMA Concentration (wt %) | 44 | 19 |
| 1. BisEMA Concentration (wt %) | 0 | 0 |
| 1. DDDMA Concentration (wt %) | 0 | 0 |
| 1. DX-511 Concentration (wt %) | 0 | 0 |
| 1. EGDMA Concentration (wt %) | 0 | 0 |
| 1. EBPDMA Concentration (wt %) | 0 | 0 |
| 1. BMAEPP Concentration (wt %) | 1 | 0.4 |
| 1. PEGDMA Concentration (wt %) | 0 | 0 |
| 1. Silorane Concentration (wt %) | 0 | 0 |
| 1. TCDUA Concentration (wt %) | 0 | 0 |
| 1. MDP Concentration (wt %) | 0 | 0 |
| 1. Filler Loading (Fill) (wt %) | 176 | 75 |
| 1. Degree of Monomer to Polymer Conversion (DoC; %) | 55 | 24 |
| 1. Viscosity (Visc; kPas) | 12 | 5 |
| 1. Depth of Cure (DepthCure; mm) | 34 | 15 |

**Appendix Table 2.** Composite Performance Outcomes (POs), Sample Sizes, Proportions to All Samples, and Whether They Were Analyzed.

| **PO** | **Sample Size** | **Proportion (%)** | **Analyzed?** |
| --- | --- | --- | --- |
| 1. Flexura Modulus (FlexMod; GPa) | 103 | 44.2 | Yes |
| 1. Flexural Strength (FlexStr; MPa) | 128 | 54.9 | Yes |
| 1. Compressive Strength (CompStr; MPa) | 42 | 18.0 | No |
| 1. Fracture Toughness (FracTough; MPa m^1/2^) | 40 | 17.2 | Yes |
| 1. Fracture Work (FracWk; kJ m^−2^) | 9 | 3.9 | No |
| 1. Polymerization Volumetric Shrinkage (ShrinkV; %) | 106 | 45.5 | Yes |
| 1. Polymerization Shrinkage Stress (ShrinkStr; MPa) | 45 | 19.3 | Yes |

**Appendix Table 3.** AI Model Performance for Flexural Modulus (FlexMod)

| **Metric** | **SVM** | **Decision Tree** | **KNN** | **LGBM** | **Random Forest** | **Logistic Regression** | **Gaussian Naïve Bayes** | **ELM** | **XGBoost** |
| --- | --- | --- | --- | --- | --- | --- | --- | --- | --- |
| **Accuracy** | 0.88 | 0.71 | **0.90** | 0.80 | 0.85 | 0.83 | 0.73 | 0.54 | 0.83 |
| **Precision** | 0.90 | 0.74 | **0.92** | 0.84 | 0.89 | 0.85 | 0.77 | 0.57 | 0.88 |
| **Recall** | 0.88 | 0.71 | **0.90** | 0.80 | 0.85 | 0.83 | 0.73 | 0.54 | 0.83 |
| **F1 score** | 0.88 | 0.71 | **0.90** | 0.80 | 0.85 | 0.82 | 0.73 | 0.53 | 0.83 |

**Appendix Table 4.** Performance Scores for Flexural Strength (FlexStr)

| **Metric** | **SVM** | **Decision Tree** | **KNN** | **LGBM** | **Random Forest** | **Logistic Regression** | **Gaussian Naïve Bayes** | **ELM** | **XGBoost** |
| --- | --- | --- | --- | --- | --- | --- | --- | --- | --- |
| **Accuracy** | 0.71 | **0.73** | 0.65 | 0.69 | 0.69 | 0.58 | 0.48 | 0.58 | 0.69 |
| **Precision** | 0.72 | **0.73** | 0.65 | 0.69 | 0.70 | 0.58 | 0.47 | 0.58 | 0.69 |
| **Recall** | 0.71 | **0.73** | 0.65 | 0.69 | 0.69 | 0.58 | 0.48 | 0.58 | 0.69 |
| **F1 score** | 0.71 | **0.73** | 0.65 | 0.69 | 0.69 | 0.57 | 0.43 | 0.58 | 0.69 |

**Appendix Table 5.** Performance Scores for Compressive Strength (CompStr)

| **Metric** | **SVM** | **Decision Tree** | **KNN** | **LGBM** | **Random Forest** | **Logistic Regression** | **Gaussian Naïve Bayes** | **ELM** | **XGBoost** |
| --- | --- | --- | --- | --- | --- | --- | --- | --- | --- |
| **Accuracy** | 0.71 | **1.00** | 0.53 | 0.71 | 0.88 | 0.76 | 0.88 | 0.76 | 0.71 |
| **Precision** | 0.85 | **1.00** | 0.58 | 0.50 | 0.88 | 0.82 | 0.90 | 0.87 | 0.85 |
| **Recall** | 0.71 | **1.00** | 0.54 | 0.71 | 0.88 | 0.76 | 0.88 | 0.77 | 0.71 |
| **F1 score** | 0.72 | **1.00** | 0.55 | 0.58 | 0.88 | 0.70 | 0.87 | 0.77 | 0.72 |

**Appendix Table 6.** Performance Scores for Fracture Toughness (FracTough)

| **Metric** | **SVM** | **Decision Tree** | **KNN** | **LGBM** | **Random Forest** | **Logistic Regression** | **Gaussian Naïve Bayes** | **ELM** | **XGBoost** |
| --- | --- | --- | --- | --- | --- | --- | --- | --- | --- |
| **Accuracy** | **0.88** | 0.63 | 0.81 | 0.75 | 1.00 | 0.75 | 0.63 | 0.56 | 0.69 |
| **Precision** | **0.89** | 0.68 | 0.85 | 0.56 | 1.00 | 0.56 | 0.68 | 0.65 | 0.66 |
| **Recall** | **0.88** | 0.63 | 0.81 | 0.75 | 1.00 | 0.75 | 0.63 | 0.56 | 0.69 |
| **F1 score** | **0.86** | 0.65 | 0.77 | 0.64 | 1.00 | 0.64 | 0.65 | 0.59 | 0.67 |

**Appendix Table 7.** Performance Scores for Polymerization Shrinkage Volume (ShrinkV)

| **Metric** | **SVM** | **Decision Tree** | **KNN** | **LGBM** | **Random Forest** | **Logistic Regression** | **Gaussian Naïve Bayes** | **ELM** | **XGBoost** |
| --- | --- | --- | --- | --- | --- | --- | --- | --- | --- |
| **Accuracy** | 0.81 | **0.81** | 0.77 | 0.74 | 0.79 | 0.79 | 0.72 | 0.65 | 0.81 |
| **Precision** | 0.81 | **0.82** | 0.77 | 0.76 | 0.79 | 0.79 | 0.73 | 0.67 | 0.81 |
| **Recall** | 0.81 | **0.81** | 0.77 | 0.74 | 0.79 | 0.79 | 0.72 | 0.65 | 0.81 |
| **F1 score** | 0.81 | **0.82** | 0.77 | 0.75 | 0.79 | 0.78 | 0.72 | 0.66 | 0.81 |

**Appendix Table 8.** Performance scores for Shrinkage Stress classification.

| **Metric** | **SVM** | **Decision Tree** | **KNN** | **LGBM** | **Random Forest** | **Logistic Regression** | **Gaussian Naïve Bayes** | **ELM** | **XGBoost** |
| --- | --- | --- | --- | --- | --- | --- | --- | --- | --- |
| **Accuracy** | **0.89** | 0.56 | 0.78 | 0.50 | 0.61 | **0.89** | 0.83 | 0.61 | **0.89** |
| **Precision** | **0.91** | 0.56 | 0.79 | 0.25 | 0.63 | **0.91** | 0.84 | 0.63 | **0.91** |
| **Recall** | **0.89** | 0.56 | 0.78 | 0.50 | 0.61 | **0.89** | 0.83 | 0.61 | **0.89** |
| **F1 score** | **0.89** | 0.55 | 0.78 | 0.33 | 0.60 | **0.89** | 0.83 | 0.60 | **0.89** |

**Appendix Table 9.** R^2^ measure for regression.

| **Model** | **FlexMod** | **FlexStr** | **ShrinkV** | **ShrinkStr** |
| --- | --- | --- | --- | --- |
| SVR | 0.83 | 0.32 | 0.72 | 0.77 |
| Decision Tree Regression | 0.77 | **0.82** | 0.74 | **0.93** |
| HistGradientBoostingRegressor | 0.84 | 0.70 | 0.79 | -0.21 |
| Random Forest Regressor | 0.77 | 0.41 | 0.77 | 0.84 |
| Voting regressor | **0.91** | 0.64 | **0.83** | 0.91 |

**Appendix Table 10.** Error of the Best Regression Models

| **Error** | **FlexMod:**  **Voting regressor (GPa)** | **ShrinkV:**  **Voting regressor (%)** | **FlexStr:**  **Decision Tree Regression (MPa)** | **ShrinkStr**  **Decision Tree Regression (MPa)** |
| --- | --- | --- | --- | --- |
| **Mean Absolute Error** | 1.08 | 0.87 | 11.86 | 0.49 |
| **Mean Squared Error** | 2.17 | 2.01 | 318.22 | 0.36 |
| **Root Mean Squared Error** | 1.47 | 1.42 | 17.83 | 0.6 |
| **Median Absolute Error** | 0.55 | 0.28 | 7.19 | 0.34 |
| **Max Error** | 3.77 | 4.07 | 55 | 1.15 |

**Appendix Table 11.** Comparison of Important CAs in Feature Importance Analysis

| **FlexMod** | | **FlexStr** | | **ShrinkV** | | **ShrinkStr** | |
| --- | --- | --- | --- | --- | --- | --- | --- |
| Classified Data | Regression Analysis | Classified Data | Regression Analysis | Classified Data | Regression Analysis | Classified Data | Regression Analysis |
| TEGDMA | TEGDMA | BisGMA | UDMA | TEGDMA | TEGDMA | BisGMA | DepthCure |
| BisGMA | DoC | DepthCure | TEGDMA | BisGMA | DoC | DepthCure | DoC |
| Fill | Fill | DoC | BisGMA | Fill | Fill | DoC | Fill |
| DepthCure | DepthCure | Fill | DepthCure | DepthCure | DepthCure | Fill | BisGMA |
| DoC | UDMA | UDMA | DoC | DoC | UDMA | UDMA |  |
| UDMA |  | TEGDMA | Fill | UDMA |  | TEGDMA |  |

**Appendix Table 12.** Error scores for Shrinkage Volume.

| **Error** | **SVR** | **Decision Tree Regression** | **Hist Gradient Boosting Regressor** | **Random Forest Regressor** | **Voting regressor** |
| --- | --- | --- | --- | --- | --- |
| **Mean Absolute Error** | 1.24 | 0.85 | 0.89 | 1.07 | 0.87 |
| **Mean Squared Error** | 3.23 | 3.06 | 2.4 | 2.65 | 2.01 |
| **Root Mean Squared Error** | 1.79 | 1.75 | 1.55 | 1.63 | 1.42 |
| **Median Absolute Error** | 0.73 | 0.13 | 0.46 | 0.62 | 0.28 |
| **Max Error** | 5.66 | 5.29 | 5.4 | 5.18 | 4.07 |

**Appendix Table 13.** Error scores for Flexural Modulus.

| **Error** | **SVR** | **Decision Tree Regression** | **Hist Gradient Boosting Regressor** | **Random Forest Regressor** | **Voting regressor** |
| --- | --- | --- | --- | --- | --- |
| **Mean Absolute Error** | 1.27 | 1.19 | 1.48 | 1.61 | 1.08 |
| **Mean Squared Error** | 3.96 | 5.19 | 3.63 | 5.37 | 2.17 |
| **Root Mean Squared Error** | 1.99 | 2.28 | 1.91 | 2.32 | 1.47 |
| **Median Absolute Error** | 0.64 | 0.4 | 1 | 1.19 | 0.55 |
| **Max Error** | 6.15 | 8.89 | 4.28 | 7.49 | 3.77 |

**Table 14.** Error scores for Flexural Strength.

| **Error** | **SVR** | **Decision Tree Regression** | **Hist Gradient Boosting Regressor** | **Random Forest Regressor** | **Voting regressor** |
| --- | --- | --- | --- | --- | --- |
| **Mean Absolute Error** | 23.14 | 11.86 | 15.32 | 22.87 | 17.02 |
| **Mean Squared Error** | 1180.08 | 318.22 | 521.12 | 1028.27 | 626.41 |
| **Root Mean Squared Error** | 34.35 | 17.83 | 22.82 | 32.07 | 25.03 |
| **Median Absolute Error** | 11.39 | 7.19 | 6.05 | 16.59 | 10.22 |
| **Max Error** | 98.64 | 55 | 0.7 | 119.46 | 93.18 |

**Table 15.** Error scores for Shrinkage Stress.

| **Error** | **SVR** | **Decision Tree Regression** | **Hist Gradient Boosting Regressor** | **Random Forest Regressor** | **Voting regressor** |
| --- | --- | --- | --- | --- | --- |
| **Mean Absolute Error** | 0.97 | 0.49 | 2.12 | 0.77 | 0.61 |
| **Mean Squared Error** | 1.19 | 0.36 | 6.25 | 0.85 | 0.47 |
| **Root Mean Squared Error** | 1.09 | 0.6 | 2.5 | 0.92 | 0.69 |
| **Median Absolute Error** | 0.92 | 0.34 | 2.61 | 0.74 | 0.53 |

# **Appendix figures**


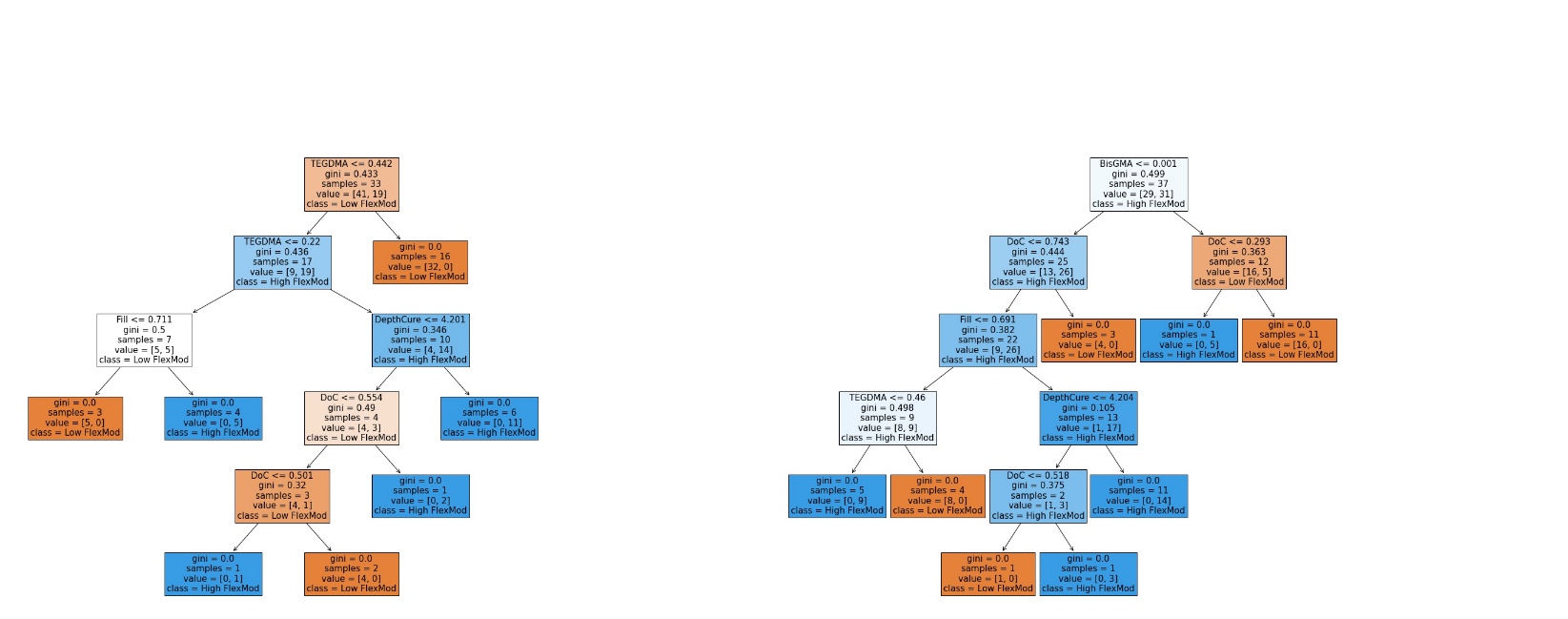


**Appendix Figure 1**. Random Forest of two the one hundred estimators, where gini is the impurity score.


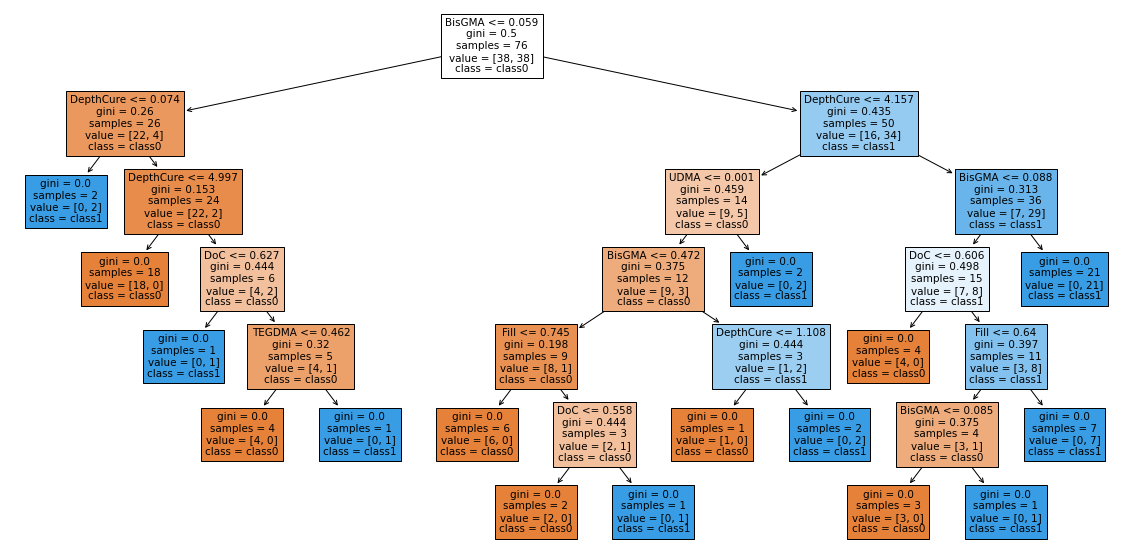


**Appendix Figure 2**. FlexStr Decision Tree. 31 nodes and Depth of 6.


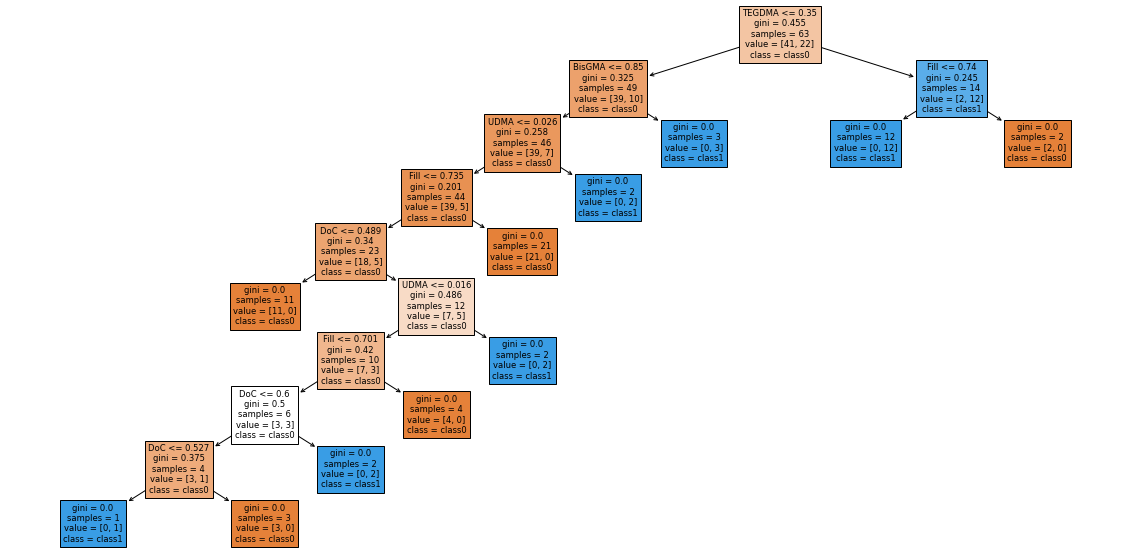


**Appendix Figure 3.** ShrinkV Decision Tree. 21 nodes and Depth of 9.
